# The Deacetylase Sirtuin 1 Regulates Human Papillomavirus Replication by Modulating Histone Acetylation and Recruitment of DNA Damage Factors NBS1 and Rad51 to Viral Genomes

**DOI:** 10.1371/journal.ppat.1005181

**Published:** 2015-09-25

**Authors:** Erika S. Langsfeld, Jason M. Bodily, Laimonis A. Laimins

**Affiliations:** 1 Department of Microbiology-Immunology, Northwestern University, Feinberg School of Medicine, Chicago, Illinois, United States of America; 2 Department of Microbiology and Immunology, Louisiana State University Health Sciences Center, Shreveport, Louisiana, United States of America; National Institute of Allergy and Infectious Diseases, National Institutes of Health, UNITED STATES

## Abstract

Human papillomaviruses (HPV) regulate their differentiation-dependent life cycles by activating a number of cellular pathways, such as the DNA damage response, through control of post-translational protein modification. Sirtuin 1 (SIRT1) is a protein deacetylase that modulates the acetylation of a number of cellular substrates, resulting in activation of pathways controlling gene expression and DNA damage repair. Our studies indicate that SIRT1 levels are increased in cells containing episomes of high-risk HPV types through the combined action of the E6 and E7 oncoproteins. Knockdown of SIRT1 in these cells with shRNAs impairs viral activities including genome maintenance, amplification and late gene transcription, with minimal effects on cellular proliferation ability. Abrogation of amplification was also seen following treatment with the SIRT1 deacetylase inhibitor, EX-527. Importantly, SIRT1 binds multiple regions of the HPV genome in undifferentiated cells, but this association is lost upon of differentiation. SIRT1 regulates the acetylation of Histone H1 (Lys26) and H4 (Lys16) bound to HPV genomes and this may contribute to regulation of viral replication and gene expression. The differentiation-dependent replication of high-risk HPVs requires activation of factors in the Ataxia Telangiectasia Mutated (ATM) pathway and SIRT1 regulates the recruitment of both NBS1 and Rad51 to the viral genomes. These observations demonstrate that SIRT1 is a critical regulator of multiple aspects of the high-risk HPV life cycle.

## Author Summary

Human papillomaviruses regulate their differentiation-dependent life cycles by activating a number of cellular pathways, such as the DNA damage response, through control of post-translational protein modification. Sirtuin 1 (SIRT1) is a protein deacetylase that regulates the acetylation of a number of cellular substrates, resulting in activation of pathways involved in gene expression and DNA damage repair. We report here that SIRT1 protein levels are elevated in cells stably maintaining genomes of oncogenic HPVs and that SIRT1 knockdown impairs genome maintenance, productive replication and late gene transcription. The DNA damage sensing and repair pathways are critical for the HPV viral life cycle and members of this pathway, such as NBS1 and Rad51, are targets of SIRT1. Our studies demonstrate that SIRT1 binds the HPV genome and regulates both viral chromatin remodeling as well as binding of members of the homologous repair pathway to viral DNA. These findings demonstrate that binding of SIRT1 to the HPV genome is necessary for histone deacetylation and recruitment of DNA damage repair factors and is a critical step in the HPV life cycle.

## Introduction

Human papillomaviruses (HPV) are small, double-stranded DNA viruses that depend upon host factors for productive replication. HPVs infect basal cells in stratified epithelia that become exposed through microwounds. Following entry, viral genomes are established as multi-copy episomes in the nuclei of infected cells. The E6 and E7 proteins provide important functions in these cells, such as inducing cell cycle progression and blocking apoptosis. As infected cells divide, daughter cells migrate away from the basal layer and undergo differentiation. In highly differentiated suprabasal layers, viral genome amplification, late gene expression and virion assembly are induced. Normal keratinocytes exit the cell cycle as they differentiate, however, HPV positive cells remain active in the cell cycle and re-enter S/G2 phases for viral amplification. This is necessary as HPV genome amplification requires the action of host cell polymerases and other replication factors. The E6 and E7 proteins are responsible for keeping suprabasal cells active in the cell cycle, as well as regulating a number of additional cellular pathways, including the ATM DNA damage response. HPV proteins constitutively activate the Ataxia Telangiectasia Mutated (ATM) pathway, which is necessary for differentiation-dependent genome amplification but not stable maintenance of genomes in undifferentiated cells [1].

The sirtuin family of proteins (SIRT1 –SIRT7) are class III histone deacetylases that utilize NAD+ as a cofactor and regulate a variety of cellular functions including response to stress, proliferation, DNA damage repair and apoptosis. In particular, SIRT1 is described as a tumor suppressor that mediates several cellular pathways in response to metabolic or genotoxic stress (reviewed in [2]). SIRT1 was originally described as *Sir2*, a histone deacetylase important for DNA damage repair and longevity in yeast [3–5]. Later studies in mammalian systems support an important role for SIRT1 in DNA damage repair, including double strand break (DSB) repair by homologous recombination [6–9]. Deacetylation of the Nijmegan Breakage Syndrome 1(NBS1) protein by SIRT1 is required for its phosphorylation by ATM and subsequent recruitment of other DNA repair factors to sites of damage [8, 10]. In addition, upon genotoxic stress, SIRT1 moves from silent promoters to sites of DNA damage, deacetylating histones H1(Lys26) and H4(Lys16), and contributing to the recruitment of DNA damage factors [9, 11–13].

HPVs target a number of pathways that regulate the acetylation of proteins, including histone deacetylases and p300, which are important for the viral life cycle [14–16]. The HPV16 E7 protein has been reported to increase the levels of SIRT1 in cervical cancer lines and this is required for enhanced survival capability [17]. Given the role of SIRT1 in regulating chromatin remodeling and activation of the DNA damage response, we hypothesized that it played a role in the differentiation-dependent HPV life cycle. Our studies indicate that knockdown of SIRT1 in HPV positive cells interferes with genome maintenance in undifferentiated keratinocytes, as well as amplification and late gene expression upon differentiation. In these cells, SIRT1 has no long-term effect on the growth or differentiation of HPV positive cells that stably maintain viral episomes. SIRT1 binds viral genomes and regulates the acetylation of bound histones, as well as recruitment of DNA damage factors NBS1 and Rad51, which is critical for the differentiation-dependent viral life cycle.

## Results

### SIRT1 protein is increased in HPV positive cell lines

**Fig 1:**
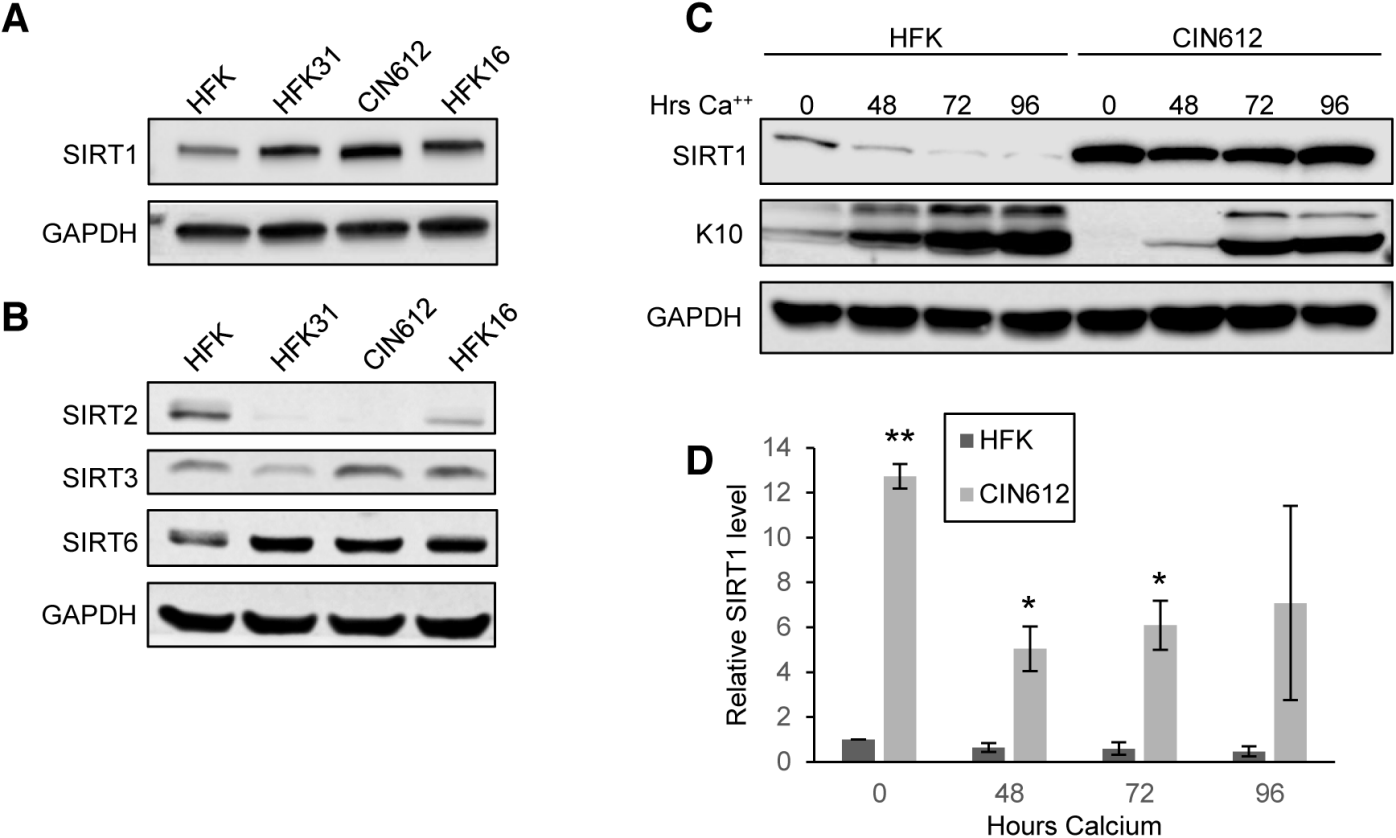
HPV induces overexpression of SIRT1 throughout keratinocyte differentiation. **(A)** Western blot analysis of SIRT1 in normal HFKs. HFKs were stably transfected with HPV 31 or 16 genomes, and CIN612 cells. **(B)** Western blot analysis of other SIRT family members in the same cells as in (A) **(C)** Western blot analysis of HFKs and CIN612 cells induced to differentiate using 1.5mM calcium chloride for up to 96 hours. Cytokeratin 10 (K10) is a marker of differentiation. **(D)** Protein blot signal intensity analysis was performed using Licor Image Studio software on results from at least three independent experiments. Error bars represent +/- 1 SEM. Significance as determined by Student’s t-test is shown as * = p<0.05, ** = p<0.005.

To investigate whether SIRT1 plays a role in the regulation of the differentiation-dependent life cycle of HPVs, the levels of SIRT1 in cell lines that stably maintain HPV 31 or 16 episomes were examined by western blot analysis. These cell lines were generated by transfecting primary human foreskin keratinocytes (HFKs) with re-circularized viral genomes and then selecting for the co-transfected drug resistance pSV2Neo marker, as previously described [18]. In addition, a cell line, CIN612, derived from a cervical intraepithelial neoplasia grade 1 (CIN I) cervical biopsy that stably maintains HPV 31 episomes was also examined. Cell lines were confirmed to maintain viral episomes by Southern blot analysis **(S1A Fig)** and examined by western blot for SIRT1 protein levels. SIRT1 protein is increased in undifferentiated monolayer cultures of HPV positive cell lines as compared to normal keratinocytes **(Fig 1A)**. To investigate whether other SIRTs are similarly increased in HPV positive cells, we tested the same lysates for sirtuins 2, 3 and 6. While no change in SIRT3 levels was detected, decreased levels of cytoplasmic sirtuin SIRT2 was observed in HPV positive cells. SIRT6 showed a similar trend to SIRT1 **(Fig 1B)**. For this study, we chose to focus on the nuclear sirtuin, SIRT1, since it is elevated in HPV containing cells and shares several targets in common with HPV.

### HPV positive keratinocytes maintain elevated levels of SIRT1 throughout differentiation

Since the life cycle of HPV is linked to the differentiation of host cells [19, 20], it was important to determine if the levels of SIRT1 proteins changed during differentiation. For this analysis, HPV 31 positive CIN612 cells were differentiated in high-calcium media, as this allows for isolation of homogeneously differentiated populations of cells as a function of time [18, 21, 22]. Viral genome amplification starts after approximately 48 hours of exposure to high-calcium media and plateaus between 72 and 96 hours. Western blots were performed on lysates collected from undifferentiated cells and from cells differentiated in high-calcium media (at 48, 72, and 96 hour time points). We observed a decline in SIRT1 protein levels as differentiation proceeded in normal HFKs **(Fig 1C and 1D)**, however, in cells containing either HPV 31 or 16 high-risk HPV types, SIRT1 protein levels remained elevated throughout differentiation **(Fig 1C and 1D and S1B Fig)**. SIRT1 also remained nuclear throughout differentiation, with a diffuse staining pattern and no preference for particular nuclear structures or foci formation **(Fig 4C and S1C Fig).** This demonstrates that HPV promotes the maintenance of high SIRT1 levels throughout differentiation and suggests a potential role in the viral life cycle.

### HPV31 E6 and E7 drive SIRT1 overexpression

**Fig 2:**
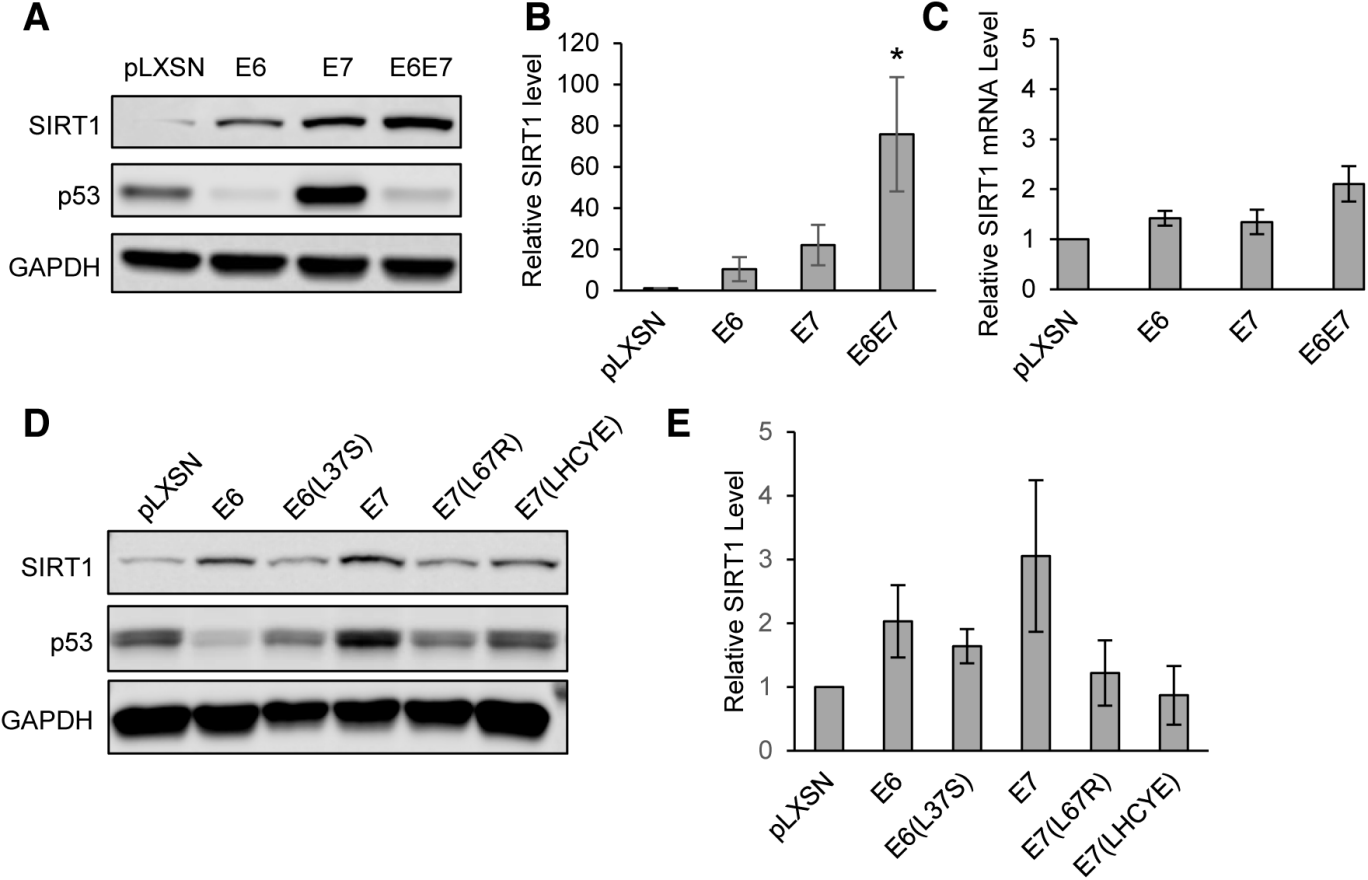
HPV31 E6 and E7 both increase levels of SIRT1 protein in primary keratinocytes. **(A)** HFK cells retrovirally transduced with either empty control vector or pLXSN encoding HPV31 E6, E7, or E6 and E7 together were stably selected with G418 and analyzed by Western blot for SIRT1. p53 protein levels was used as a positive control to confirm that E6 and E7 constructs were active. **(B)** Three independent experiments as in (A) were analyzed by Licor Image Studio software. Results were normalized to GAPDH and are expressed as fold change over pLXSN empty vector control. Error bars represent +/- 1 SEM. Significance as determined by Student’s t-test is shown as * = p<0.05 **(C)** RT-PCR performed on RNA isolated from the same cells as in (A and B). Results were normalized to signal obtained using GAPDH primers and is expressed as fold change relative to pLXSN empty vector control. Error bars represent +/- 1 SEM of the averages of 3 PCR replicates from each of 3 independent experiments. **(D)** HFK cells were retrovirally transduced with pLXSN vector control, wild-type HPV31 E6, E7, or mutants described in the text. Protein lysates were tested by western blot. **(E)** Three independent experiments, as in (D), were analyzed using Licor Image Studio software. Results were normalized to GAPDH and expressed as fold change relative to pLXSN control. Error bars represent +/- 1 SEM.

To determine if a viral protein is responsible for the observed increase in SIRT1 protein, primary keratinocytes were transduced with retroviruses expressing HPV 31 E6, E7, E6/E7, or empty vector control (pLXSN). Previous studies reported that HPV16 E7 can induce increased protein levels of SIRT1 in primary keratinocytes [17]. Stable expressing cells were isolated following G418 selection and SIRT1 protein levels analyzed by western blot. SIRT1 levels were found to be moderately increased in keratinocytes expressing either E6 or E7, with an additive effect observed upon E6 and E7 co-expression **(Fig 2A and 2B)**. To determine whether the increase in SIRT1 protein levels in HPV infected cells is due to increased transcription, qRT-PCR was performed on RNA derived from the same cells as in Fig 2A. Interestingly, while SIRT1 protein levels were substantially increased in keratinocytes expressing HPV 31 E6 and E7, mRNA levels were only modestly unregulated (approximately 2-fold) **(Fig 2A–2C)**. This indicates that elevated SIRT1 levels are the result of a post-transcriptional mechanism, presumably the result of enhanced protein stabilization or translation.

To determine which activities of E6 and E7 are responsible for SIRT1 protein accumulation, previously described mutants of HPV31 E6 (L37S) and E7 (L67R and LHCYE) were retrovirally transduced into keratinocytes. These mutants lack the ability to bind p53, histone deacetylase (HDAC), and retinoblastoma protein (Rb), respectively [14, 23]. Stably expressing lines were then selected using G418 and lysates from each were examined by western blot. [15, 23]. Each of the three mutant cell lines exhibited reduced levels of SIRT1, as compared to cells with wild type E6 or E7. The greatest effect was seen in cells expressing the E7(L67R) HDAC binding mutant or the E7(LHCYE) Rb binding mutant **(Fig 2D and 2E)**. Taken together, these data suggest that HPV31 oncoproteins E6 and E7 synergistically induce accumulation of SIRT1 protein. The p53, HDAC, and Rb binding activities of E6 and E7 all contributed to SIRT1 protein levels, however, the ability of E7 to bind HDAC or Rb showed the greatest effect. It is likely that a combination of E6 and E7 functions is required for maximal enhancement of SIRT1 levels.

### SIRT1 knockdown reduces copy number in undifferentiated cells and amplification of HPV31 genomes upon differentiation

**Fig 3:**
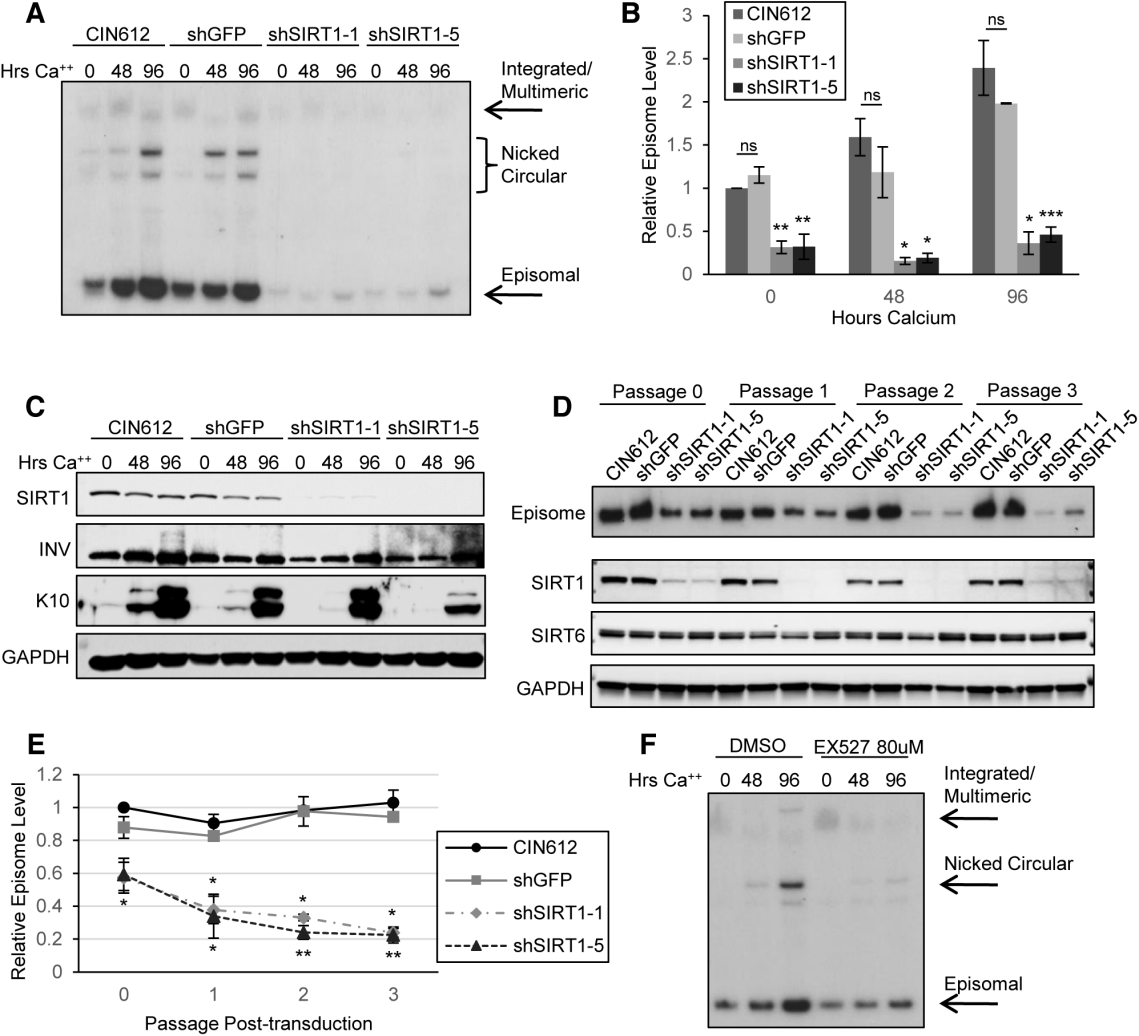
Depletion of SIRT1 by shRNA inhibits viral DNA replication and amplification (A) CIN612 cells stably transduced with shGFP control or two shSIRT1 constructs were induced to differentiate in high-calcium media for up to 96 hours. Southern blot was performed on extracted DNA from cells harvested at indicated times. (B) Signal intensity was quantified using LICOR image analysis software. Error bars indicate +/- 1 SEM of the values between at least three independent experiments. Significance of differences between shRNA knockdown cells and shGFP cells was determined by Student’s T-test and is indicated as * = p<0.05, ** = p<0.005, and *** = p<0.0005. ns = no significant difference between mock transduced and shGFP cells. (C) Protein lysates were collected from cells in (A) and Western blot analysis performed to confirm knockdown. Cytokeratin 10 (K10) serves as a marker of differentiation. Blot is representative of three independent experiments. (D) CIN612 cells were transduced with shGFP control or shSIRT1 lentiviruses and episome levels examined by Southern blot at each passage thereafter. SIRT1, SIRT6, and GAPDH protein levels were analyzed by western blot of lysates from cells taken from the same population as for the Southern blot. (E) Sub-saturated x-ray films were scanned and signal intensity was quantified using LICOR image analysis software. Episomal signal intensity at each timepoint was normalized to CIN612 Passage 0 levels. Error bars indicate +/-1 SEM of the values between at least three independent experiments. Significance was measured by Student’s t-test and is indicated as * = p < 0.05 and ** = p < 0.005. Significance for shSIRT1-1 is shown above the line and shSIRT1-5 is shown below. (F) CIN612 cells were treated for with either 80μM EX-527 or the same volume of DMSO for 48 hours, then harvested or induced to differentiate for the timepoints indicated. Southern blot was performed on DNA extracted from treated and control cells. Results shown are representative of three independent experiments.

The elevated SIRT1 levels observed in HPV positive cells suggested that it might be a critical regulator of the HPV life cycle. To determine whether increased SIRT1 is necessary for stable HPV genome replication or amplification, keratinocytes that stably maintain HPV31 genomes were transduced with lentiviruses expressing shRNAs against SIRT1 or GFP (control). Five different constructs were tested for knockdown efficiency and the two most efficient (shSIRT1_1 and shSIRT1_5) were selected for the subsequent studies **(S2A Fig)**. Cells transduced with shRNA-encoding lentiviruses were selected using puromycin and stable knockdown cell lines were isolated. Cells used for the HPV life cycle analysis experiments were passaged and analyzed between 3 and 6 passages post-transduction. Cells in which SIRT1 was stably knocked down along with passage-matched control cells, were grown in undifferentiated monolayer culture or induced to differentiate in high-calcium media for 48 and 96 hours. Total DNA was isolated from these cells and analyzed by Southern blot for levels of viral DNA. Wild-type CIN612 and shGFP control cells showed typical amplification of viral DNA upon differentiation, however, shSIRT1 cells showed dramatic impairment in both episomal genome maintenance as well as differentiation-dependent amplification in multiple HPV 31 positive cell lines **(Fig 3A and 3B and S2B Fig)**. Similar effects were seen in three independently derived knockdown lines. Knockdown of SIRT1 was maintained throughout differentiation and expression of markers of differentiation was confirmed by western blot analysis. While cells expressing shSIRT1-5 showed some impairment in cytokeratin 10 (K10) expression upon calcium-mediated differentiation, this effect was not seen in shSIRT1-1 lines, indicating that it is not specific to SIRT1 expression **(Fig 3C)**.

**Fig 4:**
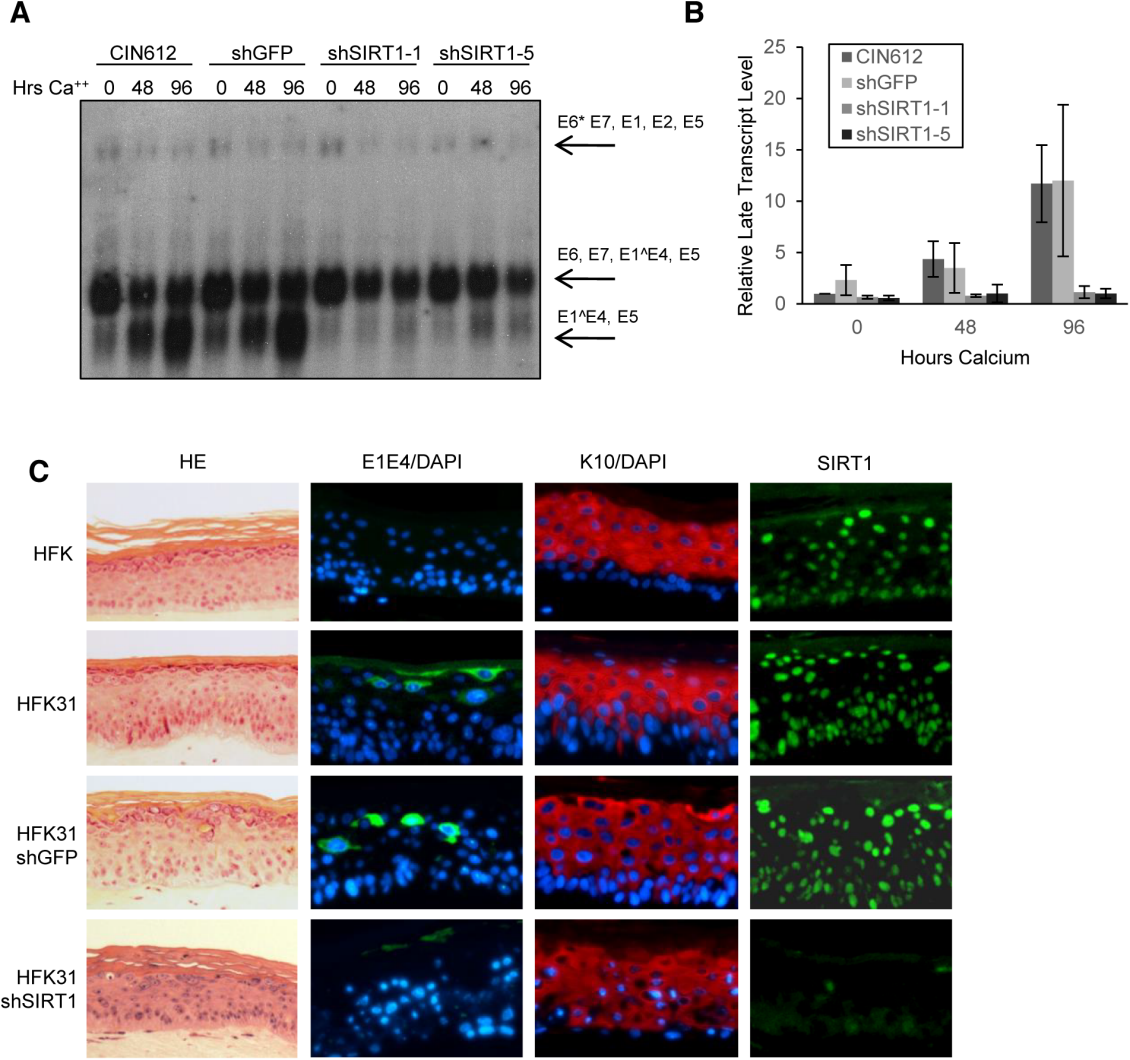
Depletion of SIRT1 by shRNAs inhibits HPV late gene transcription and expression. **(A)** Northern blot of RNA isolated from CIN612 cells stably selected to express control shRNA and shSIRT1 constructs. **(B)** Sub-saturated X-ray films from (A) were scanned and analyzed by Licor signal intensity analysis and expressed as fold change of late transcripts E1^E4 and E5 relative to CIN612 undifferentiated control. Error bars are +/- 1 SEM. **(C)** HFKs and HFKs stably expressing HPV31 and either shGFP or shSIRT1 were grown in organotypic raft culture for 14 days. HE is hematoxylin/eosin stain. E1E4 is an HPV31 late gene product and is expressed in upper layers of infected epithelium. K10 is a differentiation marker.

SIRT1 has been implicated as a longevity factor important for the continued proliferation of established cell lines (discussed in [24]). To investigate if SIRT1 knockdown has any long term effects on the proliferative ability of HPV positive cells that might indirectly affect genome replication data, the growth of knockdown cell lines was examined over multiple passages beginning after puromycin selection (approximately 7 days after transduction). Although knockdown of SIRT1 initially decreased population doubling in both normal and HPV positive keratinocytes, cells quickly recovered to near wild-type growth rates, despite sustained knockdown of SIRT1 **(S2C and S2D Fig)**. Levels of the related nuclear Sirtuin SIRT6 were not affected by SIRT1 knockdown. Cell numbers in the first 2–3 days of transduction did not significantly differ between control and SIRT1 knockdown cells, as shown by the slope of the growth curve, whereas episome numbers decreased significantly **(Fig 3E and S2E Fig)**. This observation suggests that the mechanism responsible for the observed loss of HPV genome maintenance replication is independent of the proliferative ability of the host cell DNA.

It was also important to investigate if the reduced levels of HPV genomes in stable SIRT1 knockdown lines were a direct result of a transient replicative crisis that might occur at early passages. To address this, CIN612 cells were transduced with control or shSIRT1 lentiviruses, as before, and levels of HPV episomal genomes were examined by Southern blot at each passage, until growth rates normalized. The resulting analysis shows that HPV genomes are lost early after SIRT1 knockdown, with approximately 40% of genomes lost in the initial 2–3 days after transduction, prior to any reduction in growth rate. **(Fig 3D and 3E and S2E Fig)**. Loss of HPV genomes continued steadily throughout puromycin selection (Passage 1) and subsequent passages. Since SIRT1 is known to be a mediator of escape from apoptosis and senescence, we examined levels of apoptotic markers PARP and Caspase-3 and senescence marker β-galactosidase. Activated (cleaved) forms of PARP and Caspase-3 were not detected and only minimal expression of β-galactosidase was evident in knockdown cells (**S3A and S3B Fig)**. These results indicate that the requirement for SIRT1 to maintain HPV genomes is independent of an effect on proliferation and is not due to apoptosis or senescence of SIRT1 knockdown cells.

We next investigated the specific effect of SIRT1’s deacetylase activity by examining the effects of treating CIN612 cells with the inhibitor EX-527. EX-527 is highly specific for SIRT1 versus other sirtuins with minimal effects on cell viability [25]. Cells were treated with 80 μM EX-527 or DMSO control for 48 hours and then harvested or induced to differentiate in high calcium media. Similar to the effects seen in SIRT1 shRNA transduced cells, EX-527 treated cells failed to amplify genomes upon differentiation **(Fig 3F)**. In contrast to the shRNA experiments, EX-527 had little or no effect on the stable maintenance on viral episomes in undifferentiated cells **(Fig 3A and 3F)**. To confirm specificity of EX-527 for SIRT1 and not SIRT2, we examined lysates from the same population of cells as in Fig 3F by western blot for levels of the SIRT1 target acetylated p53 and the SIRT2 target acetylated α-tubulin. The level of acetylated p53 increased markedly upon EX-527 treatment, while the level of acetylated α-tubulin remained unchanged **(S3C Fig)**. These results indicate that SIRT1 deacetylase activity is critical for HPV genome amplification upon differentiation but not for stable maintenance replication in undifferentiated cells. This suggests a non-catalytic function of SIRT1 may be important for stable maintenance of episomes in undifferentiated cells.

### Knockdown of SIRT1 impairs HPV31 late gene transcription

Transcription of HPV mRNAs is regulated during the viral life cycle by the activity of the early promoter in undifferentiated cells and the late promoter in differentiated cells [20, 26, 27]. Early transcripts encoding E6*/E7/E1^E4/E5 or E6*/E7/E1/E2/E5, as well as late transcripts encoding E1^E4/E5 can be detected by northern blot analysis. To determine whether HPV31 gene transcription is impaired upon SIRT1 shRNA knockdown, total RNA was isolated from the HPV positive cells examined in Fig 3 and analyzed by northern blot. Undifferentiated SIRT1 knockdown cells showed no significant difference in total expression of early gene transcripts as compared to control cells **(Fig 4A and 4B)**. In contrast, upon differentiation, shSIRT1 cells failed to induce late gene transcription to the levels seen in control cells. Interestingly, shSIRT1 cells continued to express high levels of early transcripts, despite a reduction in the number of viral episomes per cell, suggesting dysregulation of transcription from that promoter **(S4C Fig)**.

To address whether the defect in late viral functions observed upon calcium induced differentiation could be recapitulated in another differentiation system, HPV31 positive stable SIRT1 knockdown cells and control cells were induced to differentiate in organotypic raft cultures. This method induces full differentiation of keratinocytes into a stratified epithelium and recapitulates all stages of differentiation [18, 28]. After 14 days of differentiation, raft cultures were harvested, sectioned and stained for the late viral protein E1^E4 and the differentiation marker K10 by immunofluorescence. In multiple HPV31 positive keratinocyte backgrounds, SIRT1 knockdown resulted in reduced levels of E1^E4 expression, while K10 expression was comparable to control rafts **(Fig 4C and S4B Fig)**. The knockdown of SIRT1 was confirmed by immunofluorescence as well as western blot analysis of raft cultures **(Fig 4C and S4A Fig)**. We conclude that maintenance of SIRT1 upon differentiation of HPV positive cells is necessary for high levels of late viral gene expression.

### SIRT1 binds the HPV31 Upstream Regulatory Region (URR) and deacetylates histones

**Fig 5:**
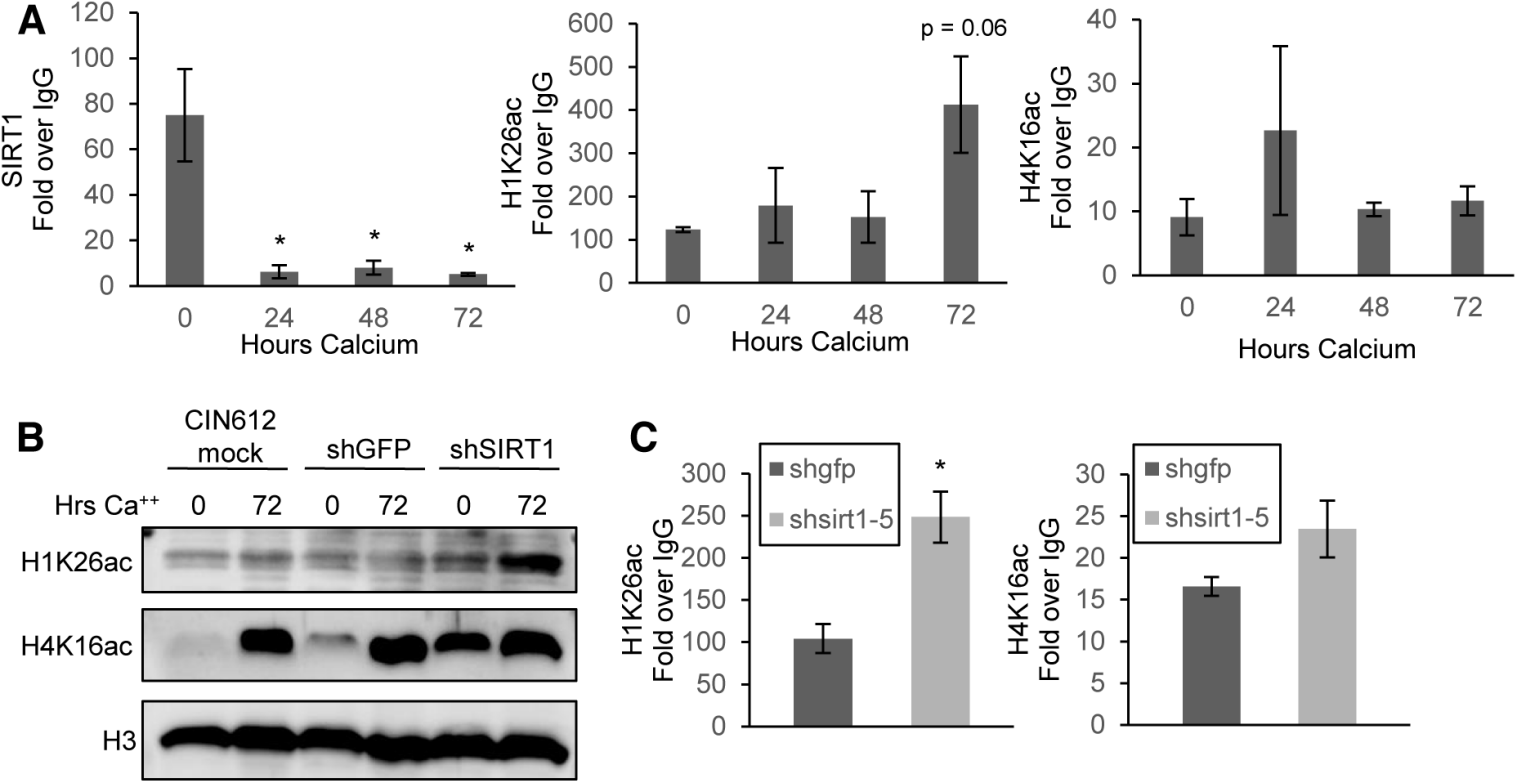
SIRT1 binds the HPV31 URR and controls histone acetylation. **(A)** CIN612 cells were grown undifferentiated in monolayer culture or induced to differentiate in high-calcium media for 72 hours and ChIP-pPCR was performed for the HPV URR. Results shown are the average Ct IP/Ct IgG using isotype matched IgG control antibody. Ct values were normalized to HPV genome input copy number. Error bars are +/- SEM of the average of three PCR replicates between three independent experiments. Significance of differences as determined by Student’s t-test is shown as * = p<0.05 **(B)** Histones were acid extracted and electrophoresed on SDS-PAGE gel. Total histone 3 (H3) serves as a loading control. **(C)** Monolayer CIN612 cells were transduced for 72 hours and ChIP-qPCR for the HPV31 URR performed as in (A). Results shown are the average fold enrichment relative to isotype matched IgG control antibody. Ct values were normalized to HPV genome input copy number. Error bars are +/- SEM of the average of three PCR replicates between three independent experiments.

Since our data indicate that SIRT1 is important for multiple steps of the HPV life cycle encompassing replication and transcription, we hypothesized that it may be acting directly on the HPV genome. We chose to focus on the Upstream Regulatory Region (URR) because it has been shown to bind DNA damage factors, transcription factors, and viral replication proteins E1 and E2 as well as contribute to replication efficiency. This region also binds histones that are regulated by acetylation during different phases of the HPV life cycle [29, 30]. SIRT1 can bind and modify histones to silence DNA and, in response to genotoxic stress, is recruited to sites of double strand breaks. We used chromatin immunoprecipitation (ChIP) assays to investigate if SIRT1 bound HPV genomes in undifferentiated and differentiated HPV genome-containing cells. We found SIRT1 bound multiple regions of the HPV31 genome and that this association was substantially reduced upon calcium-induced differentiation **(Fig 5A and S5A and S5B Fig)**. This trend was observed at all regions examined, except the L1 region.

SIRT1 has been shown to regulate acetylation of histone H1(Lys26) and H4(Lys16) [9, 11, 31]. We hypothesized that these histones would be hyperacetylated on HPV 31 DNA in differentiated cells, when SIRT1 is normally not bound. At the HPV31 URR, H1(Lys26) showed increased acetylation in 72 hour differentiated cells compared to undifferentiated cells, however H4(Lys16) did not follow a similar trend, suggesting that SIRT1 mainly controls acetylation of H1(Lys26) on the HPV genome **(Fig 5A and S5C Fig)**. This trend was also observed for H1(Lys26) at additional regions of the genome, with the most striking change observed at the HPV L2 region **(S5A Fig)**. This finding suggests that SIRT1 deacetylation of H1(Lys26) is found at multiple regions of the HPV genome and may be more pronounced at regions actively involved in replication and transcription, however, no major changes were observed in the levels of H4(Lys16) bound to the viral genome.

It was next important to determine if SIRT1 regulates total acetylation of histones or specifically targets histones bound to HPV genomes. Total histones were acid-extracted from shSIRT1 knockdown and control cells grown in monolayer or after calcium-induced differentiation. The isolates were then tested by western blot analysis for acetylation of H1(Lys26) and H4(Lys16). Depletion of SIRT1 in CIN612 cells increased the global acetylation of H1(Lys26) and H4(Lys16) and this was most pronounced in undifferentiated cells, where the levels of total acetylated H1(Lys26) and H4(Lys16) are normally low. To examine the effect of SIRT1 depletion on the acetylation of histones bound to the URR, ChIP analysis was performed on extracts of cells after 72 hour transduction with shRNAs. These studies indicate that knockdown of SIRT1 resulted in hyperacetylation of both histones bound to the URR **(Fig 5C)**. Acetylation of H1(Lys26) increased significantly while acetylation of H4(Lys16) increased only moderately. This demonstrates that deacetylation of histones bound to the HPV31 URR in undifferentiated cells is dependent upon binding of SIRT1.

### SIRT1 acts to recruit factors involved in homologous recombination to HPV genomes

**Fig 6:**
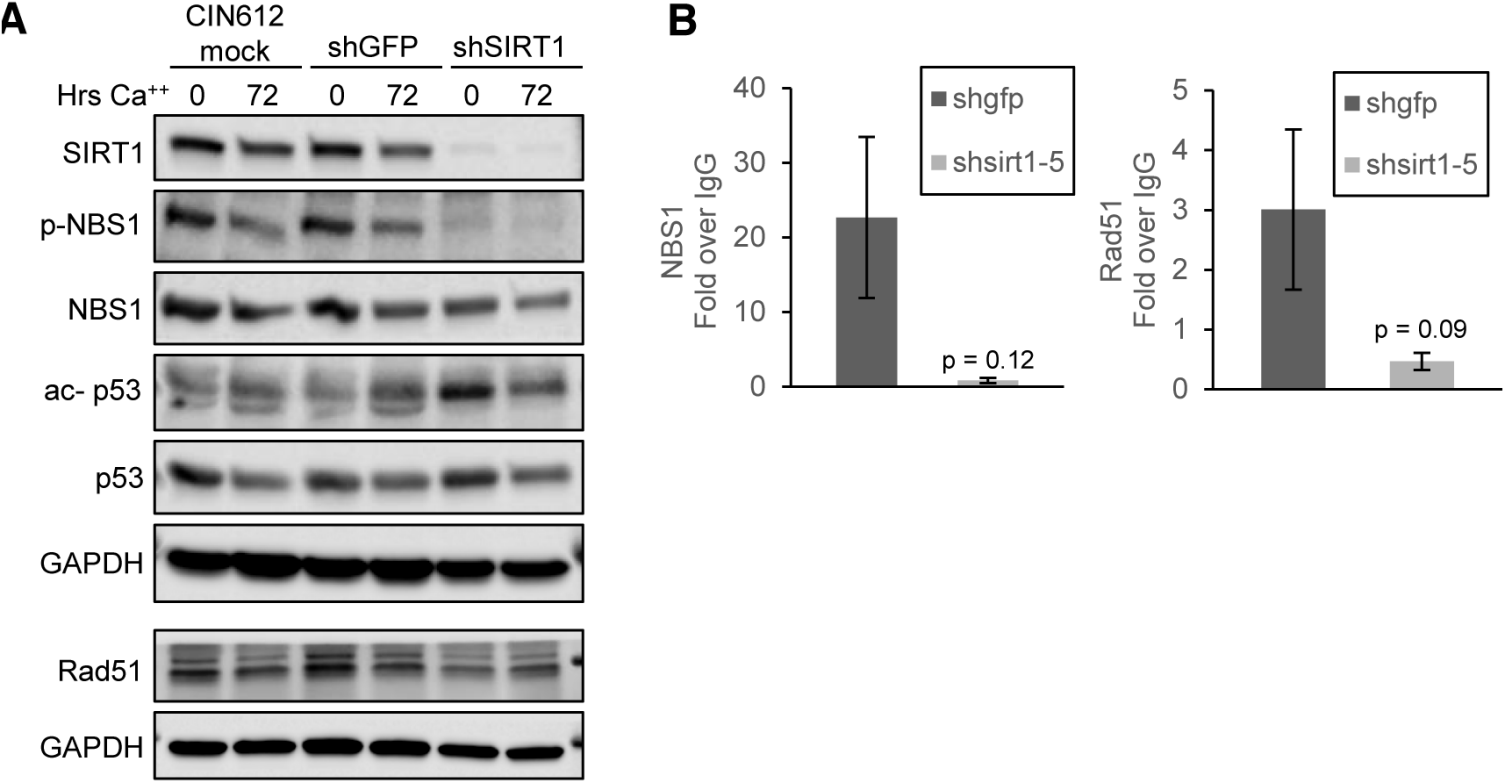
SIRT1 knockdown diminishes binding of DNA damage factors to the HPV URR. **(A)** CIN612 cells were mock transduced or transiently transduced with shGFP control or shSIRT1 for 72 hours and then induced to differentiate in high-calcium media for 72 hours. Protein lysates were analyzed by western blot. Images were captured using a Licor imaging system. **(B)** Monolayer CIN612 cells were transduced for 72 hours and ChIP-qPCR for the HPV31 URR performed. Results shown are the average fold enrichment relative to isotype matched IgG control antibody. Ct values were normalized to HPV genome input copy number. Error bars are +/- SEM of the average of three PCR replicates between three independent experiments. Significance as determined by Student’s t-test p-values are given.

The studies described above show that SIRT1 plays an important role in regulating HPV transcription and replication during the differentiation-dependent life cycle and demonstrate that these effects are mediated by binding to HPV genomic DNA and histone deacetylation activity. HPV induces an ATM DNA damage response and recruits homologous repair factors to viral genomes that are critical for differentiation-dependent amplification of HPV genomes [1]. SIRT1 regulates homologous recombination and DNA repair by directly deacetylating specific members of the ATM and ATR pathways, leading to their activation [9, 10, 32]. To investigate the effect of SIRT1 on the DNA damage response in HPV positive cells, western blot analysis of DNA damage proteins was performed on SIRT1 knockdown cells. A decrease in the level of active, phosphorylated form of NBS1 was observed, however, no changes in the levels of active, phosphorylated ATM or CHK2 were observed **(Fig 6A and S5D Fig)**. NBS1 phosphorylation requires prior deacetylation by SIRT1 and studies have shown that NBS1 activation is critical for HPV genome amplification [10, 33]. As a control an increase in the level of acetylated p53, which has been previously described, was observed in SIRT1 knockdown cells [25, 34].

Since members of the ATM DNA damage pathway are recruited to HPV genomes, we investigated whether SIRT1 affected recruitment of ATM DNA damage factors NBS1 and Rad51 to HPV 31 genomes. Cells were transduced with shGFP or shSIRT1 lentiviruses for 72 hours and the binding of NBS1 and Rad51 was examined by chromatin immunoprecipitation (ChIP). A decrease in binding of both NBS1 and Rad51 at the URR was observed relative to control cells, despite the total levels of NBS1 and Rad51 protein remaining unchanged. **(Fig 6A and 6B)**. These results support a critical role for SIRT1 in recruiting homologous recombination factors to HPV genomes and contributing to the regulation of the HPV life cycle.

## Discussion

Our findings demonstrate that SIRT1 is an important regulator of HPV DNA replication, amplification, and late gene transcription during the differentiation-dependent phase of the viral life cycle. Knockdown of SIRT1 with shRNAs interferes with the stable maintenance of viral episomes in undifferentiated cells and blocks genome amplification following differentiation. Furthermore, SIRT1 binds to multiple regions on the HPV genome and regulates the acetylation of bound histones. In addition, SIRT1 recruits homologous recombination repair factors. NBS1 and Rad51, to viral genomes, both of which are necessary for genome amplification. These studies identify SIRT1 as a critical regulator of high-risk papillomavirus replication and transcription in both undifferentiated and differentiated cells.

The levels of SIRT1 proteins are increased relative to normal keratinocytes in cells that stably maintain HPV episomes of several high-risk HPV types. In normal keratinocytes, the levels of SIRT1 rapidly decline upon differentiation, but are maintained at high levels in differentiating HPV positive cells. The ability of SIRT1 to regulate viral transcription and replication is not the result of indirect effects on cell proliferation, as our studies show that knockdown of SIRT1 has only a transient effect on the proliferative ability of HPV positive cells.

SIRT1 regulates stable maintenance of viral episomes in undifferentiated cells and knockdown reduces stable copy numbers. Knockdown of SIRT1 in cells that stably maintain HPV episomes with either of two shRNAs resulted in progressively reduced HPV genome copy numbers in undifferentiated cells, but interestingly, this did not completely abrogate episomal maintenance. We cannot, however, exclude the possibility that further passaging of knockdown cells may result in complete loss of episomes but this would not alter our conclusions that SIRT1 may be targeting a viral or cellular factor important for copy number control. Interestingly, inhibition of SIRT1 deacetylase activity by EX-527 blocks amplification but has only modest effects on maintenance replication in undifferentiated cells. This suggests that SIRT1 has additional functions beyond its deacetylase activity that are important for HPV replication. We cannot rule out, however, the possibility that the enhanced effects on replication by shRNAs are due to their more potent and specific effects as compared to drug treatment.

SIRT1 was found to bind to multiple sites on the HPV31 genome, including the URR in undifferentiated cells, and this likely contributes to regulating the level of replication. SIRT1 binding to HPV genomes is seen in undifferentiated cells, but is absent at the later stages of the life cycle. This loss of SIRT1 binding to viral DNA is, however, not due to a reduction in total levels of SIRT1, as these remain elevated in HPV positive cells upon differentiation. SIRT1 has been shown to bind chromatin at cellular promoters through its association with a variety of cellular transcription factors. In addition, following DNA damage, SIRT1 relocalizes to sites of double strand breaks and helps recruit DNA damage factors to these regions [9, 35]. Our studies show that SIRT1 binds to multiple regions of the HPV genome, likely through association with histones, members of the DNA damage repair pathway, and possibly also transcription factors. Given the dependence of amplification on an active DNA damage response, we believe that SIRT1 may be recruited to HPV genomes as part of this mechanism. This idea is supported by the observation that recruitment of DNA damage factors Rad51 and NBS1 to HPV genomes is disrupted in the absence of SIRT1.

A potential viral target of SIRT1 that could influence genome copy number control is the viral replication protein, E2, which is acetylated by p300, a known target of SIRT1 [15]. Another SIRT1 target recently shown to be important for maintenance of extrachromosomal HPV genomes is p53 [36]. In our studies, increased levels of acetylated p53 were detected in SIRT1 knockdown cells and this may impact maintenance of viral episomes. Finally, there may be additional, yet to be identified, viral or cellular proteins that are regulated by SIRT1, which are important for episomal maintenance.

Among the targets of SIRT1 that have a direct effect on amplification are members of the ATM DNA damage response, such as NBS1, whose phosphorylation is dependent on deacetylation by SIRT1 [10]. Importantly, knockdown of NBS1 with shRNAs has been shown to inhibit HPV genome amplification [33]. Our analyses demonstrate that SIRT1 regulates NBS1 phosphorylation and binding to HPV genomes. Similarly, SIRT1 regulates the binding of Rad51, a factor critical for homologous DNA repair, to viral genomes. Rad51 is recruited to HPV replication foci [37] and disruption of Rad51 activity leads to defects in homologous recombination [38]. Importantly, the levels of phosphorylated ATM and CHK2 were not decreased in SIRT1 knockdown cells and this agrees with previous studies demonstrating that recruitment of SIRT1 to sites of DNA damage is dependent on ATM activity but not the opposite [9, 10].

Our studies demonstrate that SIRT1 is bound to viral genomes in undifferentiated cells where it acts to help recruit DNA damage factors such as NBS1 and RAD51. Following binding to DNA, pNBS1, RAD51 and other DNA damage factors assemble into large complexes that are visible in replication foci. Interestingly, SIRT1 no longer remains associated with viral genomes after differentiation is initiated indicating that it is necessary for initial steps in the assembly of complexes of DNA damage factors but no longer plays an active role after these factors are bound. This is similar to E2’s role in bringing E1 proteins to viral origins leading the formation of large E1 complexes, after which E2 dissociates from viral DNAs. Overall, our data suggests that SIRT1 is an indispensable factor in the HPV life cycle that is critical for chromatin modification and initial recruitment of DNA damage factors to viral genomes.

Our analyses further show that deacetylation of histones bound to URR sequences is dependent upon SIRT1 binding to viral genomes. Depletion of SIRT1 from HPV positive cells with shRNAs, results in increased acetylation of histones H1(Lys26) and H4(Lys16) bound to the URR, with the most striking effect observed for H1(Lys26) acetylation. The finding that the pattern of global histone acetylation differed slightly from the subset of histones bound to HPV genomes is consistent with previous observations that changes to viral chromatin can occur independently of those in host chromatin [30]. Interestingly, knockdown of SIRT1 results in increased acetylation of H1(Lys26) and H4(Lys16) bound to viral genomes. This enhanced acetylation in SIRT1 knockdowns may contribute to the increased early transcription per genome observed in our studies, suggesting that SIRT1 may act as a negative regulator of early viral gene expression. In addition to the role of SIRT1 in recruiting NBS1 and Rad51 to sites of damage, mounting evidence suggests that sites of DNA damage must be prepared for repair by deacetylation of histones H1(Lys 26) and H4(Lys 16) [9, 39, 40]. We observed an increase in acetylation of these histones in the URR of HPV31 in the absence of SIRT1. This could be augmenting the effect on recruitment of NBS1 and Rad51. While acetylation of histones around the origin of replication is important for the replication of several viruses, our studies suggest that HPVs may utilize a different epigenetic mechanism to regulate the role of histones in maintenance replication.

Late viral gene transcription was also found to be impaired upon SIRT1 knockdown. Although late gene transcription is not strictly dependent on differentiation-dependent genome amplification, maximal induction can only be achieved with full amplification. Since NBS1 is required for HPV amplification, the reduction in the levels of phosphorylated NBS1, as well as loss of NBS1 binding to viral genomes upon SIRT1 knockdown, likely contributes to impaired late gene expression. Furthermore, transcription from the late promoter is dependent upon the presence of episomal copies of the HPV genome, as no transcription is seen from integrated copies [41]. Since significantly reduced levels of episomal genomes were observed in stable SIRT1 knockdown cells, this could also contribute to reduced levels of late viral transcripts. Previous studies demonstrated that upon differentiation, a DNase sensitive region around the late HPV 31 promoter is induced. This finding is consistent with an opening of chromatin around this region [30] that may be associated with a change in the acetylation state of histones. SIRT1 could function to suppress the opening of chromatin in undifferentiated cells and loss of its binding upon differentiation could allow high level replication and transcription, Surprisingly, we did not observe elevated expression of late gene transcripts in undifferentiated cells depleted of SIRT1, suggesting that other, non-histone targets of SIRT1, are required for transcription from the late promoter.

HPV 31 E6 and E7 were found to act synergistically to increase SIRT1 levels through a post-transcriptional mechanism. A previous study reported that E7 was sufficient to increase SIRT1 levels, with no effect due to E6 [17]. Our studies show a modest effect due to E6 with the most substantial increases seen with the combination of E6 and E7. Our study further shows that E6 binding to p53 contributes to SIRT1 protein overexpression, as does E7 binding to both retinoblastoma protein (Rb) and histone deacetylases (HDACs). No single mutation was sufficient to fully abrogate increased levels of SIRT1 protein, but mutation of the HDAC and Rb binding domains on HPV 31 E7 had the greatest effect. We therefore believe that the combined action of E6 and E7 is likely necessary for maximal increases in SIRT1 levels. Overall, these studies demonstrate that SIRT1 critically regulates HPV viral DNA basal replication and amplification by controlling homologous DNA damage repair factors and modifying histones bound to HPV genomes.

## Materials and Methods

### Cell Culture and generation of stable HPV transfected cell lines

Normal human foreskin keratinocytes (HFKs) were isolated from foreskins and cultured as described previously [18]. Isolated keratinocyte lines were maintained in E-Media supplemented with EGF and mitomycin-treated mouse J2 fibroblast feeder cells. CIN612 cells are an HPV31b positive line derived from a patient biopsy that was evaluated as low-grade (CIN1) [42]. HFKs were transfected with complete HPV31 genomes excised from pBR322 backbone with HindIII (New England Biosystems) and re-ligated with T4 DNA Ligase (Invitrogen). Re-circularized genomes were cotransfected into HFKs with drug resistance plasmid pSV2Neo using Fugene6 (Promega). Stably transfected clones were selected using G418. HFKs stably transfected with HPV 16 genomes were kindly donated by Ryan Hong, PhD. Before collection of cultured cells, J2 fibroblasts were removed using phosphate-buffered saline containing 0.5M EDTA (Versene) for 2 minutes followed by vigorous rinsing.

### Calcium-induced differentiation

Cells were maintained in E-media until reaching 60–80% confluence, then washed with PBS and changed to KGM media (Lonza), to wash out calcium. After 24 hours, cells were changed to KBM (Lonza) supplemented with 1.5mM CaCl_2_. After 48 hours in high-calcium, media was changed and at 48, 72 or 96 hours, cells were harvested.

### Drug inhibitor treatment

CIN612 cells were plated at approximately 5x10^5^ cells per 100mm dish and allowed to grow overnight in E media. The next day, 80μM EX-527 (Sigma) or equal volume of DMSO (Sigma) vehicle control were added to new E media and added to cells. The next day, cells to be differentiated were changed to KGM media (Lonza) to wash out calcium and after overnight incubation, were maintained in KBM media (Lonza) supplemented with 1.5mM CaCl_2_ and 80μM EX-527 or DMSO. Calcium and drug-containing media was changed every 48 hours until harvest. Undifferentiated cells were harvested after 48 hours of drug treatment.

Staurosporine was purchased from Abcam and CIN612 cells treated with 500nM or equal volume DMSO control for 4 hours to induce apoptosis.

### Stable overexpression of E6 and E7 wild-type and mutant constructs

Retroviruses were grown from PT67 cells transfected with either pLXSN plasmid or pLXSN encoding HPV31 E6, E7, or E6E7. After 72 hours transfection, PT67 viral supernatant was collected and concentrated using centrifugal concentration filters (Millipore). 100μL of the resulting retroviral supernantant was added to growth media of actively dividing, subconfluent HFKs. After 48 hours transduction, 200μg/mL G418 was added to culture medium to select neomycin resistant cell lines, then reduced to 100μg/mL after 6 days in culture. Drug selection was discontinued after all mock transduced cells had died and stable transduced lines were maintained in growth media with J2 feeder fibroblasts. Mutant E6 retroviral vector pLXSN L37S was created by site-directed mutagenesis with QuickChange II XL Site Directed Mutagenesis kit (Agilent) according to the manufacturer’s directions using the following primers: forward: GTGTGTACTGCAAGCAACAGTCACTG, reverse: CAGGTCGCAGTGACTGTTGCTTGCAG. Mutant E7 retroviral vectors have been described previously [23].

### Western blot analysis

Whole cell lysates were collected using RIPA buffer with added protease inhibitor cocktail (Roche). Insoluble fractions were collected using Urea-containing buffer. Protein was quantified using Bradford assay reagent (Biorad) and bovine serum albumin standards (Pierce). Equal amounts of protein were electrophoresed in SDS-PAGE gels and transferred to PVDF membrane Immobilon-FL (Millipore). Blots were developed using ECL-plus (GE) for 5 minutes, chemiluminescence visualized on a Licor Odyssey FL imager and quantitation done using Licor Image Studio software. All quantitated protein signals were normalized to GAPDH signal from the same gel. Antibodies used were as follows: SIRT1 (Cell Signaling), SIRT2 (Abcam), SIRT3 (Sigma), Involucrin (Santa Cruz), Cytokeratin 10(Santa Cruz), GAPDH (Santa Cruz), p-NBS1 Ser 343 (Cell Signaling), NBS1 (Santa Cruz), ac-p53 (Cell Signaling), p53 (Calbiochem), pATM Ser 1981 (Cell Signaling), ATM(Cell Signaling), pCHK2 Ser 19 (Cell Signaling), CHK2(Cell Signaling).

### Generation of shRNA lentiviral concentrate

Five lentiviral constructs targeting SIRT1 mRNA were purchased from Sigma as bacterial stocks. Stocks were grown and plasmid isolated as per the manufacturer instructions. shGFP plasmid was purchased from Addgene (plasmid 30323 from David Sabattini) [43]. 5μg of shRNA plasmid was cotransfected into 293T cells with 3.33μg Gag-Pol-Tat-Rev and 1.37μg VSVG packaging plasmids. After 72 hours, lentiviral cell supernatants were removed, spun briefly to remove cellular debris, concentrated using centrifugal filters (Millpore) and stored at -80°C. 50μL of concentrated lentivirus was used to transduce target cells.

### Generation of stably transduced shRNA cell lines

HFKs, HFK31, and CIN612 cells were transduced in 3mL E Media supplemented with EGF and 8μg/mL polybrene for 4 hours. Another 3mL E Media without polybrene was then added and incubation continued for a total of 24 hours. Media was changed the next day and cells were split if necessary. After 48 hours, 2μg/mL puromycin was added to transduced cells and mock transduced control (polybrene only). Puromycin containing media was changed every 48 hours until mock cells showed complete cell death and then stably selected cells were maintained in 1μg/mL puromycin-containing media.

### Population doubling

HFK cells with and without stably transfected HPV31 genomes as well as CIN612 cells were stably transduced as described above. After selection, 2x10^5^ passage-matched wild-type, shGFP, and shSIRT1-5 cells were plated in 60mm dishes with J2 feeder cells. When wild-type or shGFP cells reached 80% confluence, all cells were treated with Versene to remove feeders and harvested. Harvested cells were stained with Trypan Blue (BioRad) and counted using an automated cell counter (BioRad) and viability and cell number were determined. Harvested cells were serially passaged in the same manner until approximately day 30 or until natural senescence in the case of primary HFKs. Population doubling of SIRT1 knockdown and control cells was determined as previously described using the following equation:

PD = (log*Nf*–log*Ni*)/log2, where *Nf* is the number of cells counted after harvesting, and *Ni* is the number of cells seeded [44].

### Senescence-associated β-galactosidase staining

CIN612 cells and CIN612 cells stably transduced with shGFP and shSIRT1 lentiviruses were plated at low density in 6-well dishes and allowed to grow overnight it E-media. The next day, cells were rinsed with PBS, fixed and stained using the SA-β-galactosidase staining kit from Cell Signaling according to the manufacturer’s instructions. After staining, cells were overlaid with glycerol and images taken with an EVOS XL-Core microscope (Life Technologies, Carlsbad, CA) at 10x brightfield.

### Southern and northern blotting

DNA and RNA were extracted from passage-matched wild-type, shGFP, or shSIRT1 stable cell lines that were undifferentiated or induced to differentiate using high calcium media. DNA was extracted using phenol-chloroform followed by sodium acetate ethanol precipitation. Purified DNA was measured using a NanoDrop2000 (Thermo Scientific). 5μg was run on a 1% TAE gel and imaged using a GelDoc imager (BioRad). Southern blotting was done as described previously [45]. RNA was extracted using RNAStat60 reagent (Tel-Test) and purified according to manufacturer instructions. 10μg of RNA was loaded onto a 1% agarose MOPS/formaldehyde gel and imaged as above. Northern blotting was done as described previously [45].

### Organotypic raft culture and immunofluorescence staining

HFKs, HFK31 cells and CIN612 cells were stably transduced as above and 1-2x10^6^ seeded onto collagen plugs as described previously [18]. Seeded plugs were transferred to mesh grids and maintained at air-liquid interface with E-media without EGF for 14 days. Rafts were then harvested and cut into pieces from which either protein or RNA were isolated as described above or pieces that were fixed in 4% paraformaldehyde. After paraffin embedding and 4μ sectioning by the Keratinocyte Core Facility at Northwestern University, 1 slide was hematoxylin eosin stained by the Core and subsequent sections stained for either HPV E1E4 (antibody generously donated by Sally Roberts PhD, University of Birmingham), K10 (Santa Cruz), or SIRT1 (Cell Signaling) by immunofluorescence as described [46]. Slides were imaged on an EVOS(Fl) imaging system (Life Technologies, Carlsbad, CA).

### Immunofluorescence staining of high-calcium differentiated monolayer cells

CIN612 cells were plated onto #1 glass coverslips and allowed to grow overnight. Calcium differentiation was carried out as described above. Cells were rinsed with PBS, fixed in 4% methanol-free formaldehyde and stained for SIRT1 (Cell Signaling) and DAPI as described previously [47]. Slides were imaged on an EVOS(Fl) imaging system (Life Technologies).

### Histone extraction

Histones were acid-extracted from 72 hour transduced cells in monolayer or induced to differentiate using calcium using a method previously described [48]. Once isolated, 3–5μL of histones were loaded onto SDS-PAGE gel, run, and blotted onto PVDF Immobilon-FL (Millipore). Membranes were blotted using H1K26ac (Sigma), H4K16ac (Active Motif) antibodies. H3 (Abcam) antibody was used as a loading control.

### Chromatin immunoprecipitation

CIN612 cells were plated at a density of 5x10^5^ cells per 10cm dish and allowed to grow overnight in E-Media. Cells to be differentiated were moved to KGM media the next day and high-calcium media the day after as described above. For lentivirally transduced cells, the next day, cells were mock transduced (polybrene only) or transduced with either shGFP or shSIRT1-5 as described above. At 72 hours transduction, undifferentiated cells were harvested. At harvest, cells were fixed in 10mL growth media with 275μL 37% formaldehyde (Sigma, final concentration 1%) for 10 minutes at RT. Cells were then washed twice with ice cold PBS and lysed as described previously [49]. Chromatin was sheared by sonication using a BioRuptor (Diagenode, Denville, NJ). Chromatin immunoprecipitation was performed using the following antibodies: SIRT1 (Millipore), NBS1 (Abcam), Rad51 (Calbiochem), H1K26ac (Sigma), and H4K16ac (Active Motif) according to a previously established protocol[47]. Briefly, 20μL of Dynabeads (Invitrogen) were blocked with 50μL 10mg/mL BSA (Sigma) and 50μL salmon sperm DNA (Invitrogen) overnight. Antibodies were incubated with blocked beads in PBS for 6–8 hours and then antibody-bound beads were incubated with chromatin overnight. Beads were washed 8 times in RIPA buffer and eluted DNA-protein complexes reverse cross-linked for 12–16 hours at 65°C. Precipitated DNA was purified using a Gel Extraction Kit (Qiagen) and amplified using SYBR green master mix (Roche) and 480 Lite Cycler (Roche). Primers (IDT, Coralville, IA) used to amplify HPV31 DNA are as follows: URR F- 5’ GATGCAGTAGTTCTGCGGTTT 3’, URR R- 5’ TATGTTGGCAAGGTGTGTTAGG 3’, p742 F 5’ GGAGGATGTCATAGACAGTCCA 3’, p742 R- 5’ GTACCTGCTGGATCAGCCATT 3’[29], L2 F- 5’ TTTGGTGGGTTGGGTAATGG 3’, L2 R- 5’ GTAGGAGGCTGCAATACAGATG 3’[47], L1 F- 5’ GATGGGGATATGGTTGATACAGGC 3’, L1 R- 5’ TAGGGACCGATTCACCAACCGTG 3’ [29]. Results were normalized to input DNA Ct and expressed as fold enrichment over species-specific IgG binding.

## Supporting Information

S1 Fig (A) Primary keratinocytes stably transfected with HPV 16 genomes were harvested and DNA isolated and examined by Southern blot. (B) Western blot of SIRT1 levels in HFKs and HFKs stably transfected with genomes of either HPV31 or 16. Cells were induced to differentiate in high-calcium media for indicated times up to 96 hours. K10 is shown as a differentiation control and GAPDH as a loading control. (C) CIN612 cells were plated on to glass coverslips and maintained in growth media or induced to differentiate in high-calcium media for 72 hours. Cells were then fixed and stained for SIRT1 or DAPI as described in Methods.
(TIF)

S2 Fig (A) Five shRNAs were packaged into lentivirus and supernatants from transfected 293T cells were concentrated. Either 10 or 100uL were used to transduce CIN612 cells for 72 hours. Cells were harvested and protein was isolated and western blotted for SIRT1. (B) HFK cells stably maintaining HPV31 genomes stably transduced with shGFP or shSIRT1 lentiviruses were induced to differentiate in high-calcium media and DNA harvested at the indicated timepoints. Southern blot was performed on isolated DNA. (C) Results of population doubling (PD) studies performed on stable, puromycin selected control or shSIRT1 knockdown cell lines as described in Methods. Below each graph is quantitation of the PD of each experiment between control and knockdown cells in the last passage. Data are from at least two independent experiments. (D) At each passage of population doubling study in CIN612 cells, protein was isolated from remaining unseeded cells to confirm continued knockdown. (E) CIN612 cells were plated, allowed to grow overnight, then transduced with control shGFP or shSIRT1 lentiviruses. After 72 hours transduction, cells were harvested and counted. Error bars represent +/-1 SEM from at least three independent experiments.
(TIF)

S3 Fig (A) CIN612 cells from the same population as in Fig 3D and CIN612 cells treated for 4 hours with Staurosporine to induce apoptosis or DMSO vehicle control and analyzed by western blot for apoptosis markers. GAPDH serves as loading control. Results are representative of at least two independent experiments. (B) CIN612 control and SIRT1 knockdown cells were stained for senescence-associated β-galactosidase as a marker of senescence. Stained cells were imaged at 10X brightfield. Rare senescent cells are indicated with red arrows. Results are representative of at least three independent experiments. (C) CIN612 cells from the same population as in Fig 3F were analyzed by western blot for inhibition of SIRT1/2 activity. Acetylated p53 is a SIRT1 target and acetylated α-tubulin is a SIRT2 specific target. Results are representative of at least two independent experiments.
(TIF)

S4 Fig (A) HFK31 shGFP and shSIRT1 raft tissue protein was isolated and tested by Western blot to confirm SIRT1 knockdown across the raft. (B) CIN612 cells were transduced with control shGFP and shSIRT1 and stably selected with puromycin. After selection, monolayer cells were seeded onto collagen plugs and grown as organotypic rafts. Raft sections were HE stained and examined by immunofluorescence for E1E4 late protein and K10 (as a marker of differentiation). (C) Early transcript level data (E6, E7, E1^E4, E5) was normalized to episome level from southern blot of corresponding cells and expressed as fold over undifferentiated CIN612 cell control. (TIF)

S5 Fig (A) CIN612 cells were grown undifferentiated in monolayer culture or induced to differentiate in high-calcium media for 72 hours and ChIP-pPCR was performed for the HPV genomic regions indicated for SIRT1 binding. Results shown are the average Ct IP/Ct IgG using isotype matched IgG control antibody. Ct values were normalized to HPV genome input copy number. Error bars are +/- SEM of the average of three PCR replicates between three independent experiments. P values as determined by Student’s t-test are indicated. (B) HFK cells stably transfected with HPV 31 were grown undifferentiated in monolayer culture or induced to differentiate in high-calcium media for 72 hours and ChIP-pPCR was performed for the HPV31 URR SIRT1 binding as in (A). (C) CIN612 cells were grown and differentiated and ChIP performed as in (A) for acetylated H1(Lys26) and acetylated H4(Lys16). Error bars are +/- SEM of the average of three PCR replicates between at least two independent experiments. (D) CIN612 cells were mock transduced or transiently transduced with shGFP control or shSIRT1 for 72 hours and then induced to differentiate in high-calcium media for 72 hours. Protein lysates were analyzed by western blot. Images were captured using a Licor imaging system. Signal was quantitated using Licor Image Studio software and each sample normalized to signal for GAPDH. Values are given relative to CIN612 control cells at 0 hours calcium.
(TIF)
