# Supplementary figures and images for: The Deacetylase Sirtuin 1 Regulates Human Papillomavirus Replication by Modulating Histone Acetylation and Recruitment of DNA Damage Factors NBS1 and Rad51 to Viral Genomes
Source: PLoS Pathog. 2015 Sep 25;11(9):e1005181. doi: 10.1371/journal.ppat.1005181 (PMC4583417; doi:10.1371/journal.ppat.1005181)

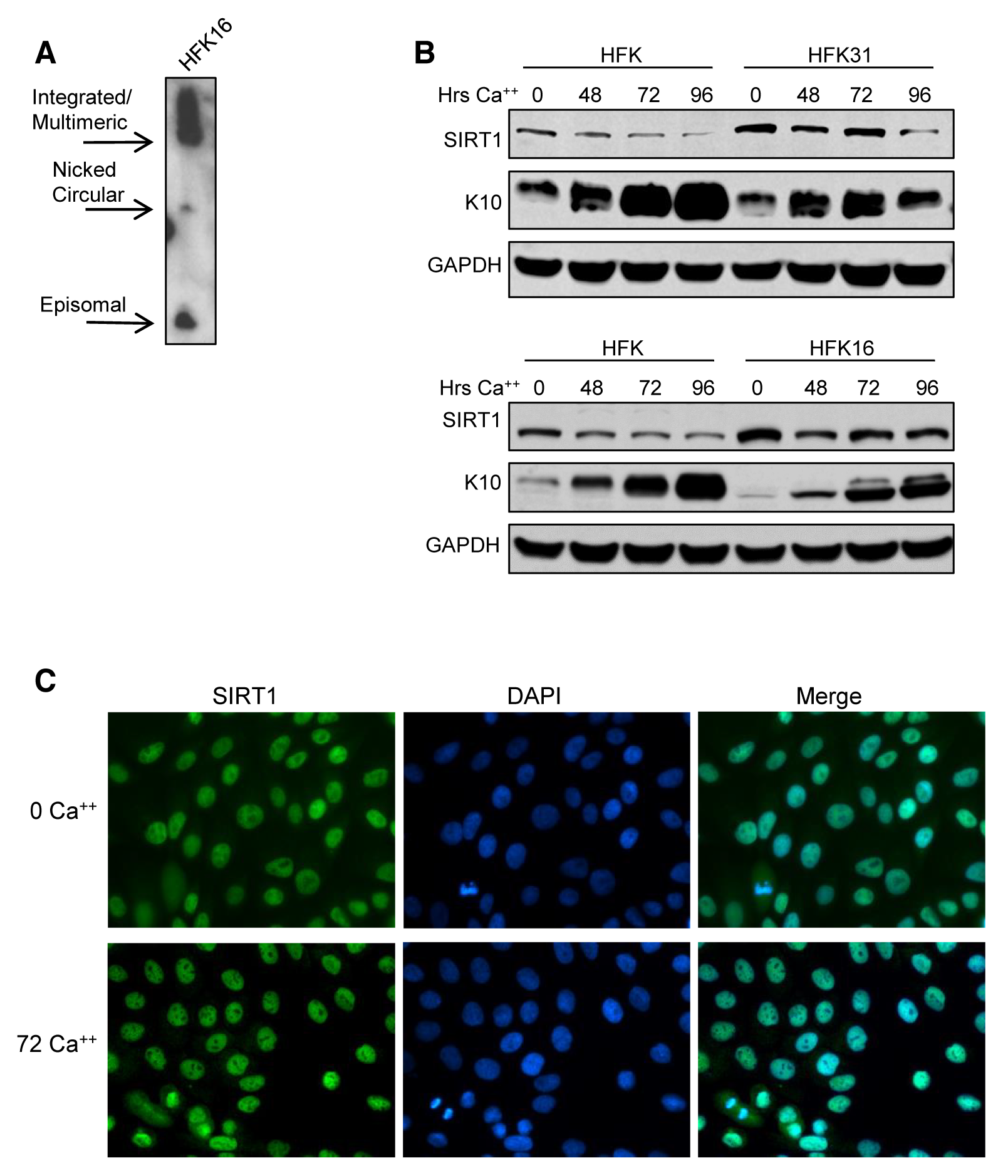

Supplement: S1 Fig — Cells were then fixed and stained for SIRT1 or DAPI as described in Methods. (TIF) [file ppat.1005181.s001.tif]

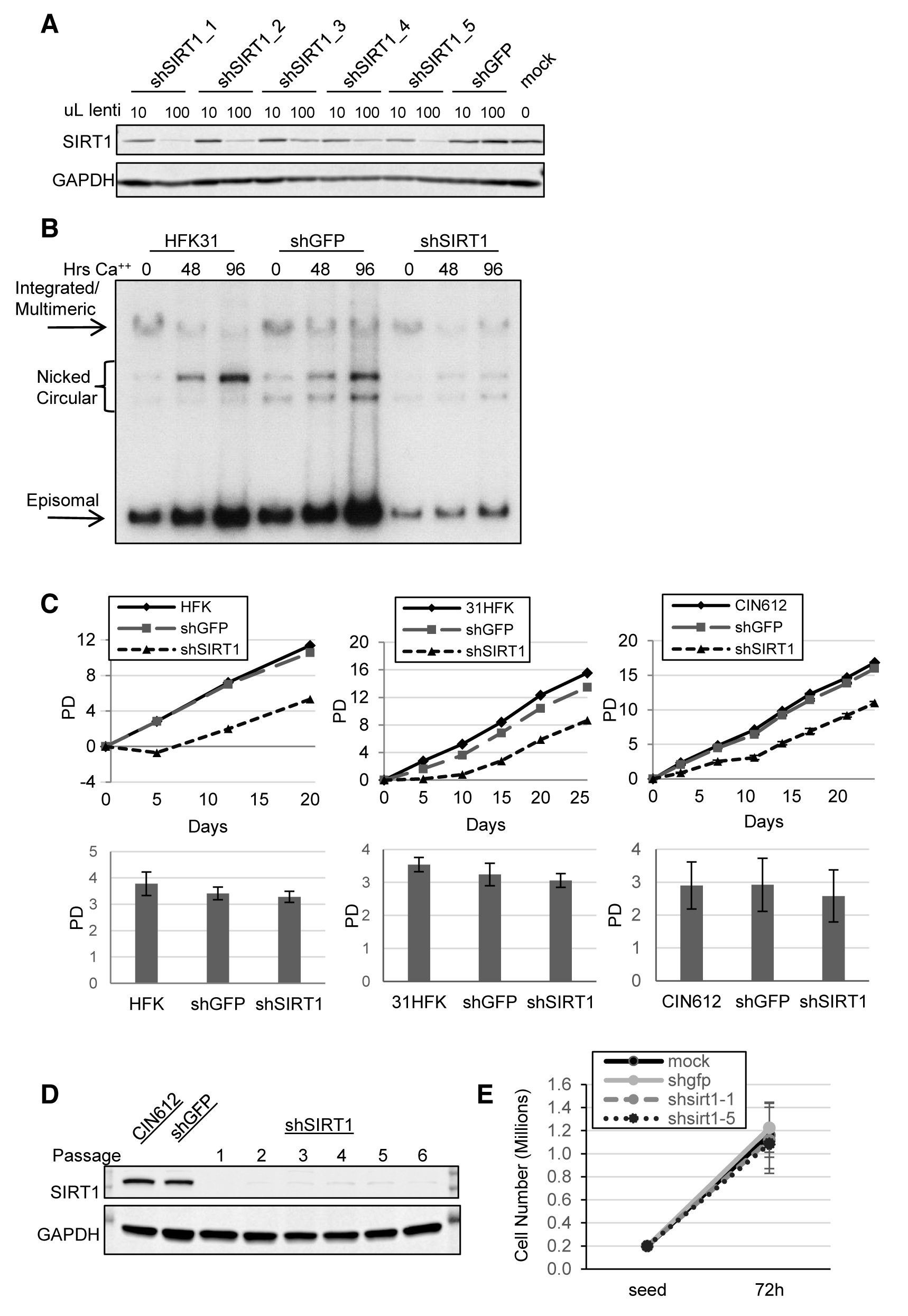

Supplement: S2 Fig — After 72 hours transduction, cells were harvested and counted. Error bars represent +/-1 SEM from at least three independent experiments. (TIF) [file ppat.1005181.s002.tif]

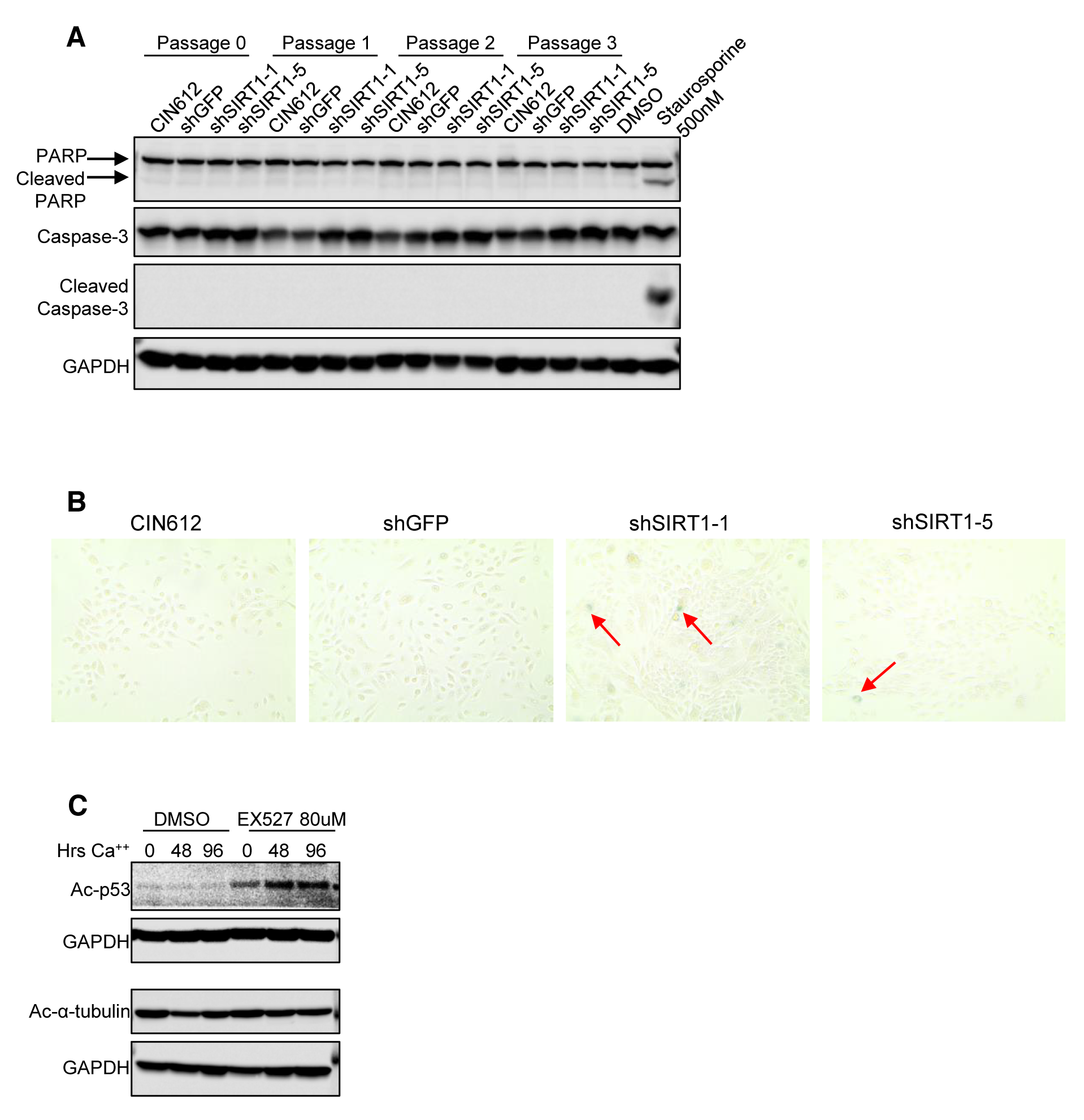

Supplement: S3 Fig — Acetylated p53 is a SIRT1 target and acetylated α-tubulin is a SIRT2 specific target. Results are representative of at least two independent experiments. (TIF) [file ppat.1005181.s003.tif]

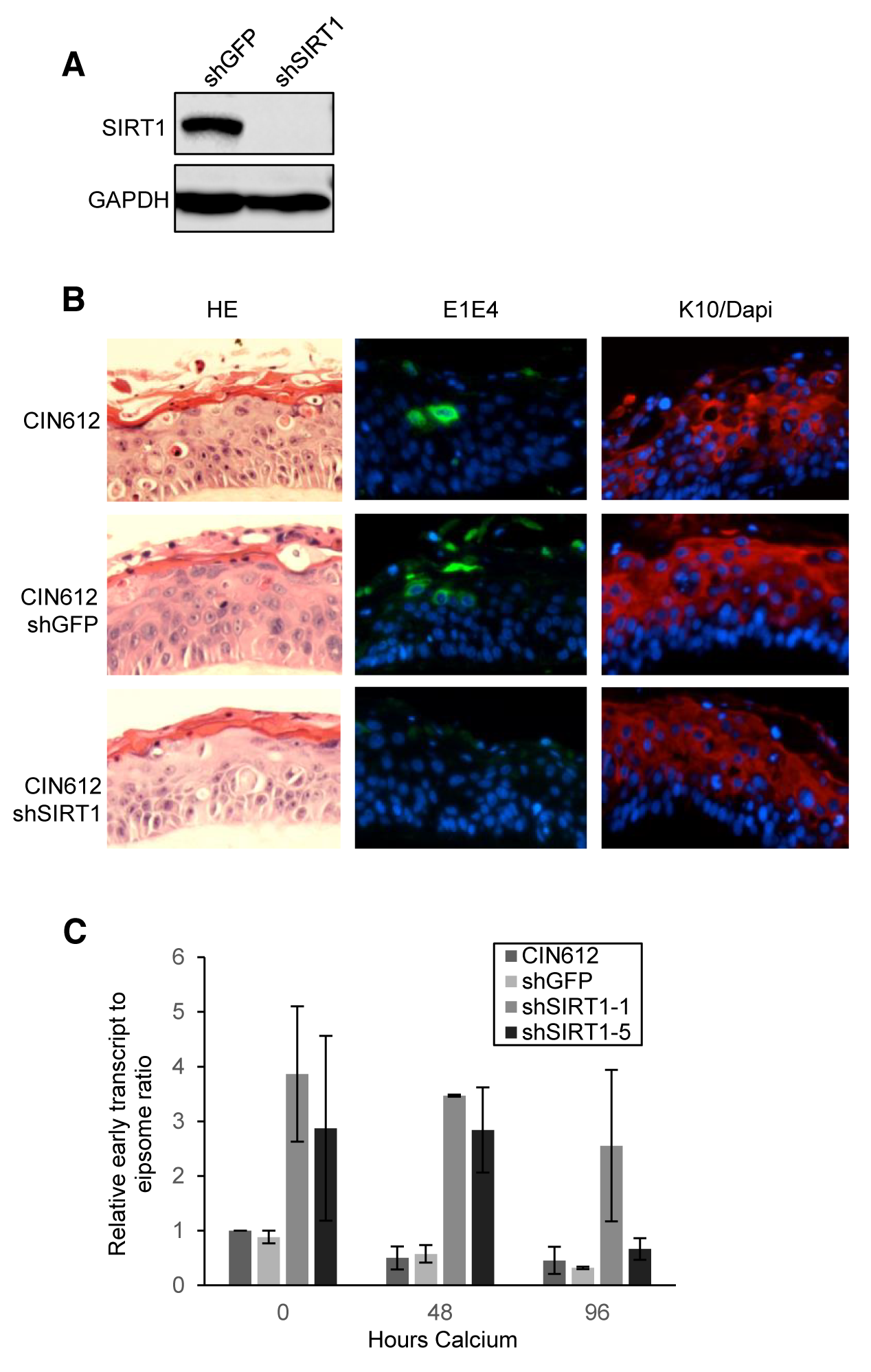

Supplement: S4 Fig — (TIF) [file ppat.1005181.s004.tif]

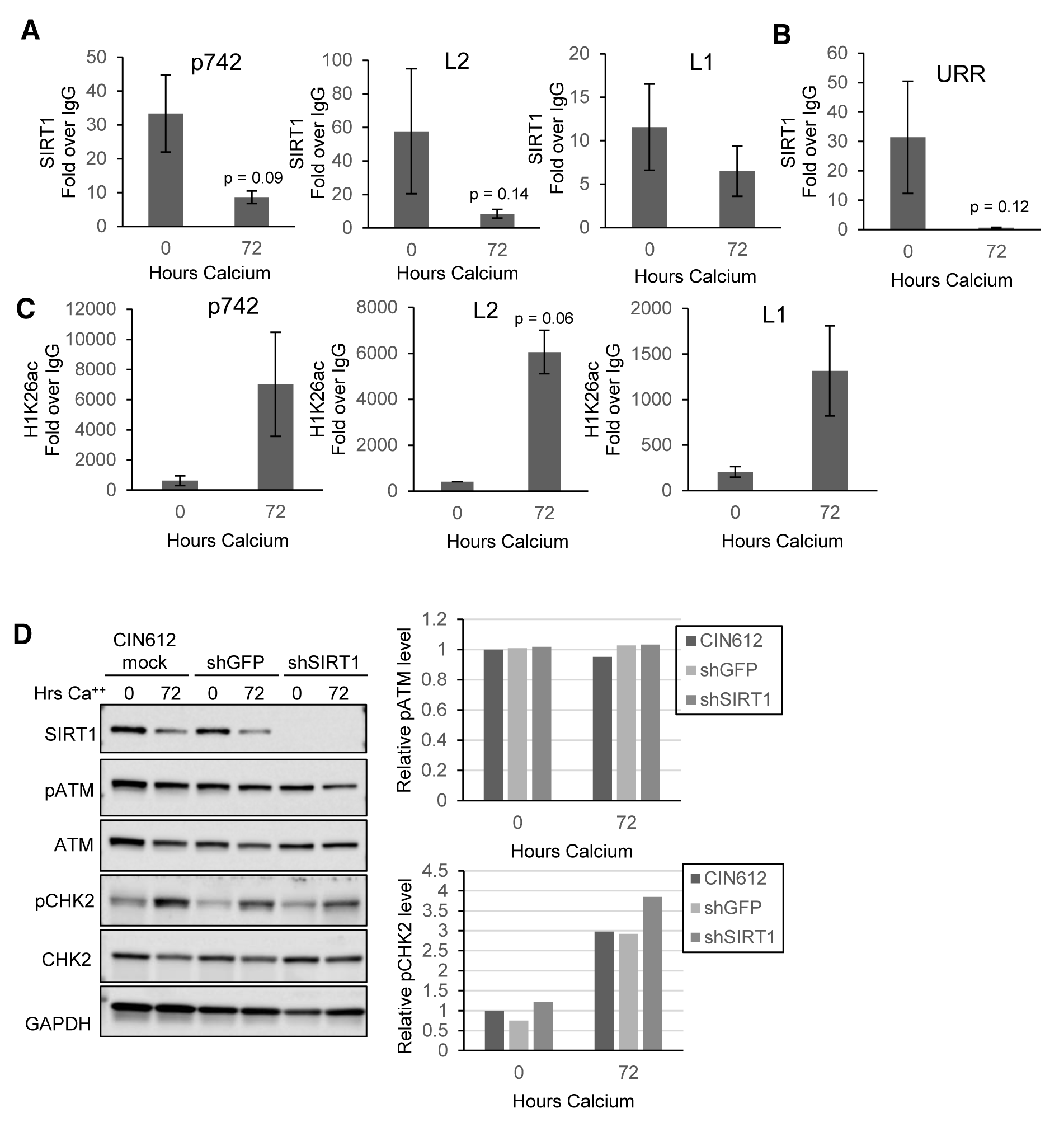

Supplement: S5 Fig — Protein lysates were analyzed by western blot. Images were captured using a Licor imaging system. Signal was quantitated using Licor Image Studio software and each sample normalized to signal for GAPDH. Values are given relative to CIN612 control cells at 0 hours calcium. (TIF) [file ppat.1005181.s005.tif]
